# Investigation of the stresses on pterygoid and zygomatic implants used in atrophic maxilla rehabilitation by finite element analysis

**DOI:** 10.4317/medoral.27199

**Published:** 2025-05-27

**Authors:** Ufuk Bakay, Belgin Gulsun, Ridvan Guler

**Affiliations:** 1 Department of Oral and Maxillofacial Surgery, Faculty of Dentistry, Dicle University, Diyarbakir, Turkiye; 2 Professor, Department of Oral and Maxillofacial Surgery, Faculty of Dentistry, Dicle University, Diyarbakir, Turkiye; 3 Assistant Professor, Department of Oral and Maxillofacial Surgery, Faculty of Dentistry, Dicle University, Diyarbakir, Turkiye

## Abstract

**Background:**

This study aims to compare stresses from vertical and oblique forces on pterygoid and zygomatic implants, combined with dental implants, in a atrophic maxilla using finite element stress analysis.

**Material and Methods:**

A computed tomography scan was used to create a geometric model of a completely edentulous adult maxilla. The maxillary bone was scanned using cone beam computed tomography (ILUMA, Orthocad, CBCT, 3M Imtec, Oklahoma, USA), and the obtained sections were transferred to the 3D-Doctor (Able Software Corp., MA, USA) software. Two models were created in the study. In the first model, zygomatic and dental implants were used, while in the second model, pterygoid and dental implants were used. A 150 N vertical force and a 100 N oblique force at a 30-degree buccolingual angle were applied. With finite element analysis assessed stress distribution in the implants and peri-implant bone tissue.

**Results:**

When the obtained stress data were examined, under vertical forces, the maximum stress on the implants was higher in Model 1 (151.984 MPa) compared to Model 2 (151.773 MPa), but no significant difference was observed. The stress formed in the metal substructure was higher in Model 2 (422.042 MPa) compared to Model 1 (308.376 MPa). The maximum principal stress in the alveolar bone was greater in Model 2 (46.866 MPa) compared to Model 1 (15.719 MPa), and the minimum principal stress in the alveolar bone was also greater in Model 2 (80.360 MPa) compared to Model 1 (76.310 MPa). Under oblique forces, the average stress on the implants was higher in Model 2 (128.297 MPa) than in Model 1 (79.607 MPa).

**Conclusions:**

When the stresses occurring on zygomatic and pterygoid implants and the alveolar bone surrounding these implants were compared, it was observed that the use of zygomatic implants was more beneficial in reducing both dental and biomechanical stress.

** Key words:**Atrophic maxilla, dental implants, finite element stress analysis, pterygoid implants, zygomatic implants.

## Introduction

The first research on implantology began in the 1960s (1). Implant procedures have successfully overcome challenges arising from anatomical issues and are now considered a successful option for rehabilitation (2,3). The goal of dental implant therapy is to apply implants and implant-supported fixed prostheses to patients, ideally within the same session, and to restore both function and aesthetics in the shortest possible time (4).

The application of dental implants in the posterior maxillary region is challenging for several reasons when there is severe atrophy, and the osteointegration period for implants in this area is longer (5). The traditional surgical procedures for advanced maxillary atrophy are sinus lift or augmentation methods using titanium mesh-supported or iliac bone-derived cancellous bone grafts. However, these methods have disadvantages such as complexity, potential morbidity at the recipient site, the need for hospitalization, increased costs, the inability to provide temporary prostheses during the graft healing process, prolonged healing times due to grafting, and a higher risk of infection especially in sinus lift procedures. Alternatively, there are options such as inlay/onlay grafts, guided bone regeneration, distraction osteogenesis, Le Fort I interpositional grafts, the use of angled implants, zygomatic implants, and pterygoid implants (6-9).

Zygomatic implants (ZI) offer a reliable and predicTable treatment option as an alternative to more invasive methods (6-10). Compared to other techniques, ZI provide several advantages, including lower cost, fewer complications, and quicker completion of prosthetic rehabilitation (6). However, some complications associated with ZI can be observed. The main complications include sinusitis, intraoral soft tissue infection, oroantral fistula, facial-periosteal hematoma, gingival hyperplasia, infraorbital paresthesia, penetration and perforation of the orbital cavity, prosthesis fit issues, temporary sensory nerve disorders, moderate nasal bleeding, subcutaneous malar emphysema, and peri-implant soft tissue infection (7).

Pterygoid implants (PI) are successfully used in the treatment of atrophic jaws (8). These implants are generally sTable and enable the rehabilitation of atrophic or resorbed posterior maxilla without the need for sinus lifting or bone grafting. In 1992, a researcher named Tulasne suggested that the pterygomaxillary region could be an alternative implantation site to avoid grafting procedures and sinus augmentation in an atrophic maxilla, and he chose this region for implant applications (11).

Finite Element Analysis (FEA) is a method used to transform a complex geometric structure into a mesh structure to analyze changes caused by force. The structure is divided into finite elements connected by nodes. The type, arrangement, and number of these elements affect the outcome of the analysis. Stress and displacement at each node can be calculated. The FEA method has also been used to examine the interaction between dental implants and bone, providing valuable information for clinical applications (12-14).

Although pterygoid implants have become a popular treatment in recent years, there are not many studies comparing their biomechanical behavior with zygomatic implants. The aim of this study is to use FEA to examine and compare the stresses caused by vertical and oblique forces on pterygoid and zygomatic implants, applied in combination with dental implants, in a severely atrophic maxilla and their impact on surrounding tissues.

## Material and Methods

- Creation of three-dimensional models in finite element analysis

Since the study was conducted using finite element analysis, ethics committee approval is not required. In this study, 3-dimensional (3D) finite element models of the maxillary bone, zygomatic bone, implant fixtures, and superstructure were used to evaluate the amount and distribution of stress in the implants and surrounding cortical and trabecular bone. A 3D model of the maxillary and zygomatic bone was developed from computed tomography (CT) image datasets of a completely edentulous patient with severe maxillary bone resorption (ILUMA, Orthocad, CBCT, 3 M Imtec, Oklahoma, USA). Following reconstruction of volumetric data with a slice thickness of 0.2 mm, the slices were exported in Digital Imaging and Communications in Medicine (DICOM) 3.0 format. Bone tissue was separated according to Hounsfield values ​​by interactive segmentation method using 3D-Doctor software (Able Software Corp., MA, USA), and after segmentation, the 3D model was obtained by 3D complex rendering method.

The implants and prosthesis components used in the study were scanned with a SmartOptics 3D scanner. The acquired models were saved in .stl format and imported into Rhinoceros 4.0 software (3670 Woodland Park Ave N, Seattle, WA 98103, USA). Using Rhinoceros software, the Boolean method was used to ensure appropriate adaptations between the bone tissue and the upper and lower parts of the prosthesis and to optimize force transmission.

- Integration of systems

To ensure the desired functionality of the finite element stress analysis program and to obtain accurate results, the elements used in the system must be specifically defined for the analysis program. In this study, the elements defined in the system include the zygomatic bones, maxillary bones, zygomatic implants, pterygoid implants, dental implants, abutments, metal substructures, and prosthetic superstructures. In our research, the connections between the implant-supporting tissue, implant-abutment, and abutment-implant-metal substructure were seamless, ensuring proper load transfer. It was determined that the implants used in the model had 100% osseointegration with the bone tissue.

- Material properties

All models were considered to be linear, homogeneous, and isotropic materials. A homogeneous material means that its mechanical properties are similar in every structural element. Isotropy refers to the property of materials having the same characteristics in all directions. Linear elasticity indicates that the deformation of the structure changes proportionally to the applied forces. For the modeling of trabecular bone, the material properties of D4 bone were used. Titanium was chosen as the material for the metal substructure. The elastic modulus and Poisson ratio values of each structure constituting the models were obtained from the literature and shown in Table 1 (15,16).

- Creation of models

In the scope of the research, two different model conFigurations were planned: In Model 1, one zygomatic implant was planned for each jaw, along with one dental implant each in the incisor lateral and first premolar tooth regions. Zygomatic implants were designed according to the extrasinus technique. In Model 2, one pterygoid implant was planned for each jaw, along with one dental implant each in the incisor lateral and first premolar tooth regions.

In our study, zygomatic implants (Nobel Biocare Zigoma implant) with a diameter of 4 mm and length of 35 mm, dental implants (Nobel Biocare Active implant) with a diameter of 3.5 mm and length of 10 mm, and pterygoid implants (Nobel Branemark Mk 3 Groovy Pterygoid implant) with a diameter of 4 mm and length of 15 mm were applied. The upper and lower jawbones and the superstructure were fixed to have zero displacement and/or rotation in each degree of freedom (DOF). In each model, the loading area was selected to mimic the contacts during chewing. In the all models created, a vertical force of 150 N and an oblique force of 100 N at a 30-degree angle were applied to tooth regions 2-4-6-7. The analyses conducted measured the stresses in the maxillary alveolar bone as maximum and minimum principal stresses, and the stresses in the implants and metal substructure as Von Mises stresses in megapascal (MPa) (N/mm²). During the analysis, high-stress areas were shown in red, while low-stress areas were marked in blue.

Cortical bone, trabecular bone, prosthetic units, and implants were transferred into the model to reflect their full morphology. The modeling process was completed by placing the models in accurate coordinates in 3D space using Rhinoceros 4.0 software and VRMesh (VirtualGrid Inc, Bellevue City WA, USA). The models were then transferred in .stl format to Algor Fempro (ALGOR, Inc., 150 Beta Drive, Pittsburgh, PA 15238-2932 USA) for analysis. During the meshing process, the models were created as much as possible using 8-node brick elements. Regions closer to the center of the structures in the models used fewer node elements. To facilitate the analysis process, vertical and narrow regions in the models were adjusted by removing linear elements. A mesh convergence test with 5% tolerance was applied to ensure mesh size and element count. Table 2 shows the number of elements and nodes used for all models.

Using 3D finite element analysis, von Mises stresses on the implants, as well as the maximum and minimum principal stress values of cortical and trabecular bone adjacent to the implants, were calculated. For stress analysis, von Mises stresses for dental implants and maximum (tensile) and minimum (compressive) principal stresses for peri-implant cortical and trabecular bone were calculated (17). The highest stress values were determined by selecting the node with the maximum value for each structure. To automate the calculation of stress values, the software's range, color, and magnitude scales were used. Von Mises, tensile, and compressive stress values were represented with a color diagram ranging from red to red. In the images evaluating von Mises and tensile stress values, red areas represented high-stress regions, and the colors transitioned to green and blue as the stress decreased. In the images showing compressive stress, blue areas represented high-stress regions, and the colors transitioned to red as the stress decreased.

## Results

- Findings Related to Von Mises Stress Values on Implants, Metal Substructures, and Alveolar Bone Under Vertical Forces

The maximum Von Mises stress values occurring in the implants under vertical loading are shown in Table 3. The maximum Von Mises stress values caused by the applied vertical forces on the implants were measured as follows: In the first model, the highest stress value was found to be 151.984 MPa in the posterior zygomatic implant. In the second model, the highest stress value was analyzed as 151.772 MPa in the premolar region dental implant. In both Model 1 and Model 2, the lowest stress value was found in the anterior dental implant, with stress values of 78.821 MPa and 76.014 MPa, respectively. Additionally, it was observed that as the number of implants increased, the Von Mises stress values on the dental implants decreased. When both models were examined, and the stress values on the dental implants were evaluated according to vertical forces, it was observed that the most suiTable planning was in Model 2.

**Figure 1:**
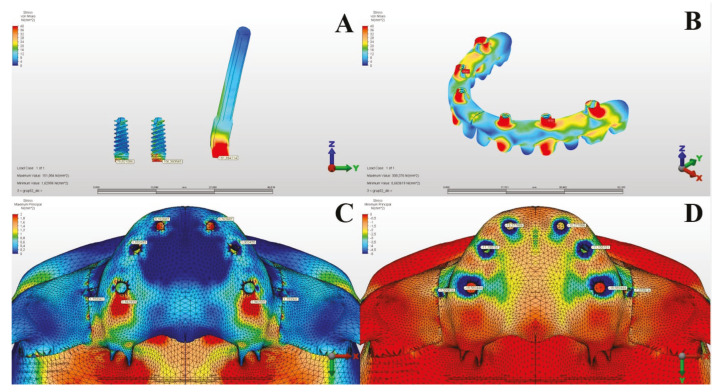
In Model 1, under Vertical Forces: Implants (A), Metal Substructure (B), Maxillary Alveolar Bone Maximum (C), and Minimum (D) Stress Values.

**Figure 2:**
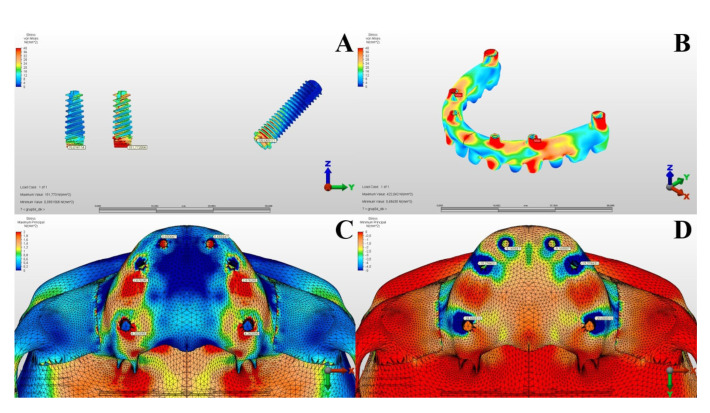
In Model 2, under Vertical Forces: Implants (A), Metal Substructure (B), Maxillary Alveolar Bone Maximum (C), and Minimum (D) Stress Values.

The Von Mises stress values in the metal substructures caused by vertical forces were measured as 308.376 MPa in the first model and 422.042 MPa in the second model. When both models were examined and the stress values ​​occurring in the metal substructures were evaluated according to the vertical forces, it was seen that the most appropriate planning was in Model 2 (Table 3, Table 4) (Fig. 1, Fig. 2).

According to the results of the vertical forces, the Von Mises values in the alveolar bone were measured as 15.719 MPa in the first model and 46.866 MPa in the second model. In Model 2, it was observed that the stress increased on the alveolar bone located distal to the pterygoid implant, around the implant in the anterior, and posterior to the implant in the premolar region. Considering the maximum principal stress values ​​in the alveolar bone under vertical forces in all groups, it was determined that the most appropriate planning was Model 1, which applied dental implants to one lateral and one premolar tooth region in addition to one zygomatic implant.

The minimum principal stress values in the alveolar bone caused by vertical forces were measured as 76.310 MPa in the first model and 80.360 MPa in the second model. According to the minimum principal stress values ​​occurring in the alveolar bone as a result of vertical forces, it was observed that Model 2 had higher stress values ​​than Model 1 (Table 3, Table 4) (Fig. 1, Fig. 2).

According to the results of our study, although the maximum Von Mises stresses on the implants against vertical forces are close to each other, it was observed that they were higher in Model 1. When examining the maximum and minimum principal values in the metal substructure and the alveolar bone, it was found that the stress was greater in Model 2. It was observed that the stress areas concentrated in the neck regions of the implants and the stresses in Model 2 were distributed more homogeneously.

- Findings Related to Von Mises Stress Values on Implants, Metal Substructures, and Alveolar Bone Under Oblique Forces

The maximum Von Mises stress values occurring in the implants under vertical loading are shown in Table 3. The maximum Von Mises stress values caused by the applied vertical forces on the implants were measured as follows: In the first model, the highest stress value was found to be 79.607 MPa in the dental implant in the premolar region. In the second model, the highest stress value was similarly analyzed as 128.296 MPa in the dental implant in the premolar region. In both Model 1 and Model 2, the lowest stress value was found in the anterior dental implant, with stress values of 62.573 MPa and 763.681 MPa, respectively. When both models were examined, and the stress values on the dental implants were evaluated according to oblique forces, it was observed that the most suiTable planning was in Model 1.

**Figure 3:**
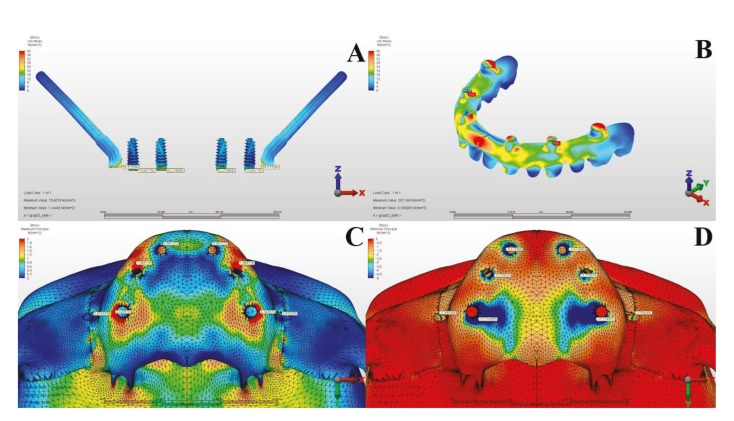
In Model 1, under Oblique Forces: Implants (A), Metal Substructure (B), Maxillary Alveolar Bone Maximum (C), and Minimum (D) Stress Values.

**Figure 4:**
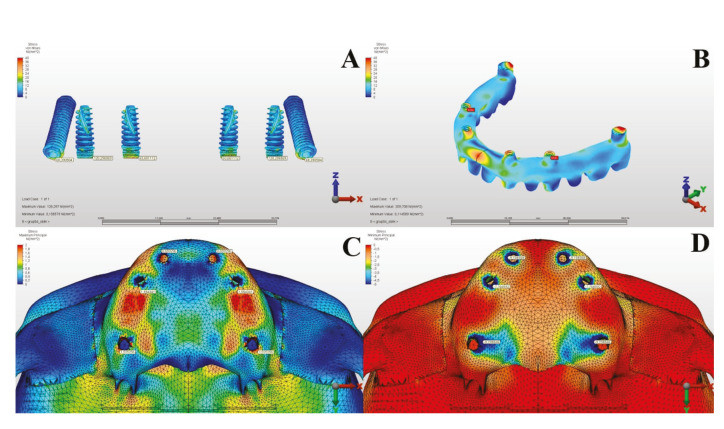
In Model 2, under Oblique Forces: Implants (A), Metal Substructure (B), Maxillary Alveolar Bone Maximum (C), and Minimum (D) Stress Values.

The Von Mises values in the metal substructures caused by oblique forces were measured as 257.148 MPa in the first model and 209.708 MPa in the second model. When both models were examined, and the stress values in the metal substructures were evaluated according to oblique forces, it was observed that the most suiTable planning was in Model 2 (Table 3, Table 4) (Fig. 3, Fig. 4).

According to the results of the oblique forces, the maximum principal stress values in the alveolar bone were measured as 12.640 MPa in the first model and 13.111 MPa in the second model. In Model 2, the stress was measured as the highest in the neck region of the implant in the premolar region, while it was determined that the stress on the posterior pterygoid implant was greater than the stress affecting the neck region of the anterior dental implant. When the maximum principal stress values occurring in the alveolar bone under oblique forces were considered across all groups, it was determined that the most suiTable planning was in Model 1, where a zygomatic implant was applied in addition to a dental implant in the lateral and premolar tooth regions.

The minimum principal stress values in the alveolar bone caused by oblique forces were measured as 40.624 MPa in the first model and 69.298 MPa in the second model. According to the minimum principal stress values occurring in the alveolar bone as a result of oblique forces, it was observed that the stress value in Model 2 was higher than in Model 1 (Table 3, Table 4) (Fig. 3, Fig. 4).

When evaluating the stress values in the implants under oblique forces across all models, it was observed that the most ideal planning was in Model 1, where a zygomatic implant was placed along with an additional incisor lateral and a dental implant in the premolar region. It was noted that the stress areas concentrated in the neck regions of the implants and that the stresses on the implants in Model 1 were less accumulated and more homogeneously distributed compared to Model 2.

## Discussion

Advanced surgical procedures for the rehabilitation of completely edentulous patients lead to an increase in both treatment duration and costs. Additionally, this situation results in reduced postoperative patient comfort and extended healing times, which increase the risk of complications. To mitigate these disadvantages, alternative treatment options are being utilized in contemporary dental practice (18).

Maxillary sinus augmentation is a valid technique that can be used to place implants in the maxillary bone when there is insufficient vertical bone height. However, this technique can lead to various complications. Notably, the most common complications include Schneiderian membrane perforation, intraoperative bleeding, infraorbital nerve injury, orbital wall perforation, displacement of the implant within the sinus, edema, infection of the placed graft, flap dehiscence, and fistula formation (19).

During the literature review, no data were found comparing the biomechanical behavior of the stresses created by the Zygomatic and Pterygoid implant systems on the alveolar bone, implant and metal substructures in the reconstruction of the atrophic maxilla. In 2023, Varghese *et al*. aimed to compare the stress distribution in 2 different zygomatic implant treatment methods by finite element analysis. In the first group, they used two zygomatic implant on one side; in the second group, they used one zygomatic implant and one conventional implant. The models were loaded with a vertical force of 150 N, a lateral force of 50 N, and a distributed occlusal force of 300 N applied to the insertion area of the masseter muscle. A difference in distribution pattern was observed when the models were loaded without the muscle component applied. The maximum deformation of the bones surrounding the implants occurred at the abutment connection of the conventional anterior implant in the model with an additional conventional anterior implant (20).

In 2014, Wen and colleagues conducted finite element stress analysis studies using the Brånemark technique, as well as extra-sinus and extra-maxillary techniques, with various numbers of zygomatic implants and different numbers and locations of dental implants. They created 9 different models for this study and applied 150 N vertical and 50 N lateral forces to these models to compare stress values. The study found that the group with bilateral zygomatic implants using the extra-sinus technique and bilateral lateral dental implants experienced the lowest stress levels (15).

Akay and colleagues evaluated three different implant-retained obturator prostheses in three models: Model 1, which included one zygomatic implant (ZI) and one dental implant (DI), Model 2, which included one ZI and two DIs, and Model 3, which included two ZIs. As a result of this study, they reported that using two ZIs on both sides of the maxilla was advantageous compared to placing DIs. Various studies on this topic have reported that the use of ZIs generally reduces the amount of stress in non-defective regions, while increasing the number of DIs reduces stress distribution to a lesser extent (21). In a 2024 finite element method (FEM) study, Gümrükçü and colleagues compared the biomechanics of six zygomatic implants and four ZIs combined with dental implants in different maxillary defects. As a result of this study, the highest stress values in the bone and implants were found in the most distal implant regions. In various studies, the maximum von Mises stress values were reported to be located in the neck regions of distal implants, both in our study and in this study (22).

In 2010, Miyamoto *et al*. obtained a threedimensional solid model in a computer environment using the CT of a patient who had hemimaxillectomy. They applied two zygoma implants to the maxillectomy side and 2 and 3 conventional dental implants to the unaffected side. They reported that the application of zygomatic implants to the affected side reduces the stresses on the prosthetic superstructure and that the forces are evenly distributed (23). In our study, in parallel with the literature results, we observed that the stress in Model 1, which included one zygomatic implant on each side and two dental implants in the anterior region, was lower and distributed more homogenously across the implant and alveolar bone.

In a study conducted in 2024, Daniel and Pande performed an *in vitro* finite element study on a three-dimensional model of zygomatic and pterygoid implants. In this study, which included a total of 24 implants, two bilateral zygomatic and pterygoid implants and two anterior dental implants were placed in the models. Vertical forces of 150 N and lateral forces of 300 N were applied to the models. As a result of the study, it was observed that pterygoid implants exhibited higher stress concentration compared to zygomatic implants (24).

Pterygoid implants, first described by researcher Tulasne in 1992, were initially used to provide anchorage from the posterior region of the atrophic maxilla. Their primary aim was to improve axial loading by eliminating posterior cantilever and to eliminate the need for grafts.11 In a clinical study conducted by Candel and colleagues in 2012, they reported an approximate success rate of 90.7% for 1,053 pterygoid implants placed in 676 patients. They concluded that pterygoid implants, which exhibit a level of bone loss similar to conventional implants, are an effective treatment option for the rehabilitation of the posterior maxilla (25). In a systematic review conducted by Bai and colleagues in 2022, they reported a success rate of 94.87% for 1,983 pterygoid implants placed in 634 patients (26).

Stefanelli and colleagues, in a case series involving 14 patients, placed 2-4 implants in the anterior region between the maxillary sinuses and 2 implants in the pterygoid process area, naming this treatment protocol the "Da Vinci Bridge." They used a dynamic navigation system for the placement of the pterygoid implants in their study. As a result, it was stated that the use of pterygoid implants in full-arch rehabilitation of the maxilla shortened the treatment duration and was a valid option for performing minimally invasive surgery (27).

Wilkirson and colleagues, in a study conducted in 2021, created six models with different implant positions and numbers to simulate complete edentulism of the maxilla. Maximum stress and deformation in the pterygoid implants and surrounding bone under occlusal forces were observed in Model 4, which included two pterygoid implants and two anterior implants. The researchers concluded that pterygoid implants reduced the levels of stress and deformation in the surrounding bone in all examined conditions (28).

In a finite element stress analysis study conducted by Daniel and colleagues in 2024, they investigated and compared the biomechanics of zygomatic and pterygoid implants in atrophic maxilla with three different bone types. The results indicated that the stress generated on pterygoid implants was greater than that on zygomatic implants (24). In our study, we observed that the stress on Model 2, which included one pterygoid implant on each side along with one incisor lateral and one premolar dental implant on each side, was greater compared to Model 1, which used zygomatic and dental implants. However, the data we obtained indicated that pterygoid implants still provided successful results in the rehabilitation of atrophic maxilla.

The present study has several limitations due to the nature of finite element models. Firstly, 100% osseointegration was assumed between the implants and the surrounding bone. However, in clinical situations, the rate of osseointegration may decrease due to factors such as infection, medications, and metabolic diseases. Another factor is that the applied load in the study is static, whereas in real-life situations, the load applied during mastication may vary due to differences in muscle force, bone shape, and complex jaw and temporomandibular joint movements. Although anatomical structures and chewing forces were optimally simulated, the study was conducted under *in vitro* conditions, limiting the full reflection of oral conditions. In finite element analysis, high-value red areas represent permanent deformation of the material. However, this is applicable to solid models, not soft or hard vital tissues. According to Frost's theory, the results of this study can be interpreted as indicating that the regions with the highest stress values are those most prone to early resorption (29). However, there is no definitive conclusion that resorption occurs in areas where the highest stress occurs. Additionally, various simplifications, including the assumption that cortical and trabecular bone are homogeneous and isotropic, were made in the present study. However, in a clinical scenario, bone anisotropy is a well-known significant factor affecting stress and strain in peri-implant bone.

Overall, the aforementioned limitations applied to all models evaluated in this study, as the primary aim was to compare stresses in different implant conFigurations rather than to provide absolute values. Despite the limitations, this study is useful and can provide predictions for clinicians in a biomechanical sense prior to zygomatic and pterygoid applications. Therefore, long-term clinical studies or three-dimensional finite element analyses are necessary to determine the effects of observed stress levels on the functionality of tissues and prostheses.

## Conclusions

In patients with bilateral atrophic maxilla, the use of zygomatic and pterygoid implants for implant-supported prosthetic rehabilitation showed that both surgical planning models, Model 1 and Model 2, yielded successful results based on the stress values obtained. It was observed that the stress values were higher in Model 2, where pterygoid implants and dental implants were used. Combining zygomatic implants with dental implants may be beneficial in reducing biomechanical stress around both dental and zygomatic implants. Considering these unpredicTable conditions and FEA results during the surgical stage, the use of zygomatic and pterygoid implants should be supported by clinical studies and long-term follow-ups should be conducted.

## Figures and Tables

**Table 1:** Elastic modulus and Poisson's ratios of the materials used in all models.

Materıals	Young Modulus (MPa)	Poisson Ratio
Cortical	13700	0.30
Cancellous	1370	0.30
Titanium (implant, abutment, screw)	110000	0.35
Cobalt-Chromium (substructure)	218000	0.33
PMMA (prosthesis)	3000	0.35
Sinus	14000	0.30

**Table 2:** Total number of elements and nodes used in models.

Models	Number of Elements	Number of Nodes
Model 1	1446550	337053
Model 2	1670894	379656

**Table 3:** Maximum Von Mises stress values of implants in models under vertical and oblique forces.

Forces		Model 1	Model 2
Vertical Forces	Anterior Implant	78.821 MPa	76.014 MPa
	Posterior Implant	106.360 MPa	151.772 MPa
	Zygomatic Implant	151.984 MPa	-
	Pterygoid Implant	-	106.065 Mpa
Oblique Forces	Anterior Implant	62.573 MPa	63.681 MPa
	Posterior Implant	79.607 MPa	128.296 MPa
	Zygomatic Implant	69.573 MPa	-
	Pterygoid Implant	-	68.390 MPa

**Table 4:** Tension amounts created as a result of vertical and oblique forces.

Forces	Models	Max Von Mises Stress Values ​​of Implants in Models	Max Von Mises Stress Values ​​for Metal Substructure	Maximum Principal Stress Values ​​of Alveolar Bone	Minimum Principal Stress Values ​​of Alveolar Bone
Vertical Forces	Model 1	151.984 MPa	308.376 MPa	15.719 MPa	76.310 MPa
	Model 2	151.773 MPa	422.042 MPa	46.866 MPa	80.360 MPa
Oblique Forces	Model 1	79.607 MPa	257.148 MPa	12.640 MPa	40.624 MPa
	Model 2	128.297 MPa	209.708 MPa	13.111 MPa	69.298 MPa
